# Supplementary material for: Complex‐centric proteome profiling by SEC‐SWATH‐MS
Source: Mol Syst Biol. 2019 Jan 14;15(1):e8438. doi: 10.15252/msb.20188438 (PMC6346213; doi:10.15252/msb.20188438)
Supplement: Supplementary file 8 — Dataset EV7 [file MSB-15-e8438-s008.zip › feature_plots_string/Q9UQL6.pdf]

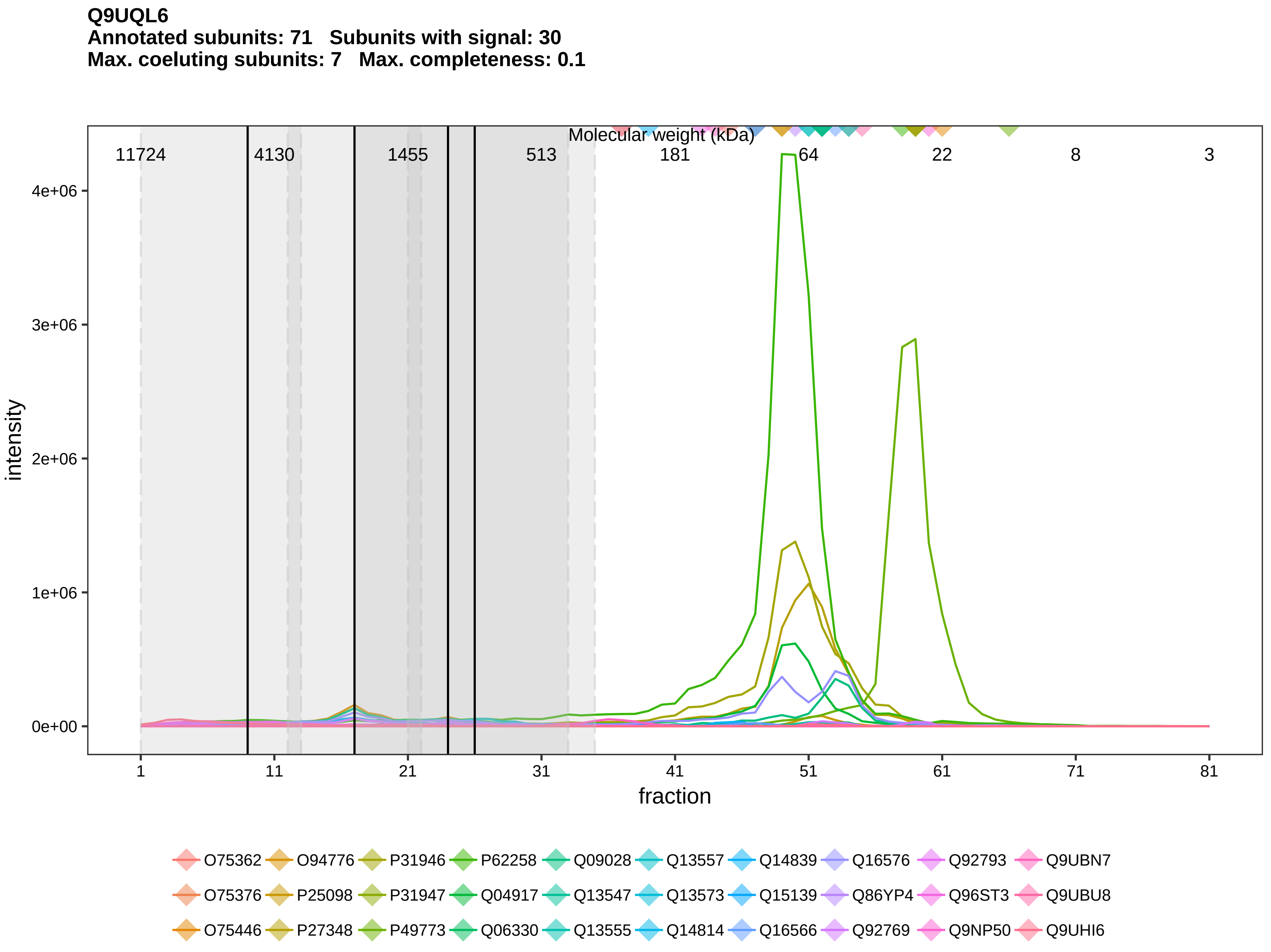
Q9UQL6
Annotated subunits: 71 Subunits with signal: 30
Max. coeluting subunits: 7 Max. completeness: 0.1
Molecular weight (kDa)
11724
4130
1455
513
181
64
22
8
3
4e+06
3e+06
2e+06
1e+06
0e+00
intensity
1
11
21
31
41
51
61
71
81
fraction
O75362
O94776
P31946
P62258
Q09028
Q13557
Q14839
Q16576
Q92793
Q9UBN7
O75376
P25098
P31947
Q04917
Q13547
Q13573
Q15139
Q86YP4
Q96ST3
Q9UBU8
O75446
P27348
P49773
Q06330
Q13555
Q14814
Q16566
Q92769
Q9NP50
Q9UHI6
